# Role of histamine H_4_ receptor in the anti-inflammatory pathway of glucocorticoid-induced leucin zipper (GILZ) in a model of lung fibrosis

**DOI:** 10.1007/s00011-023-01802-3

**Published:** 2023-10-10

**Authors:** Silvia Sgambellone, Marta Febo, Mariaconcetta Durante, Silvia Marri, Serafina Villano, Oxana Bereshchenko, Graziella Migliorati, Emanuela Masini, Carlo Riccardi, Stefano Bruscoli, Laura Lucarini

**Affiliations:** 1 Section of Pharmacology, Department of Neuroscience, Psychology, Drug Research and Child Health (NEUROFARBA), University of Florence, Viale Gaetano Pieraccini, 6, 50139 Florence, Italy; 2 Section of Pharmacology, Department of Medicine and Surgery, University of Perugia, Piazzale Severi, 1 06132 S. Andrea Delle Fratte, Perugia, Italy; 3 Department of Philosophy, Social Sciences and Education, University of Perugia, 06100 Perugia, Italy

**Keywords:** Pulmonary fibrosis, Glucocorticoids, GILZ, Histaminergic system, Inflammation

## Abstract

**Introduction:**

This study investigates the interactions between histaminergic system and glucocorticoid-induced leucin zipper (GILZ) in the inflammatory process and glucocorticoid modulation in lung fibrosis.

**Methods:**

Wild-type (WT) and GILZ Knock-Out (KO) mice were treated with bleomycin (0.05 IU) or saline, delivered by intra-tracheal injection. After surgery, mice received a continuous infusion of JNJ7777120 (JNJ, 2 mg/kg b.wt.) or vehicle for 21 days. Lung function was studied by measuring airway resistance to air insufflation through the analysis of pressure at airway opening (PAO). Lung samples were collected to evaluate the expression of histamine H_4_R, Anx-A1, and p65-NF-kB, the activity of myeloperoxidase (MPO), and the production of pro-inflammatory cytokines.

**Results:**

Airway fibrosis and remodeling were assessed by measuring TGF-β production and α-SMA deposition. JNJ reduces PAO in WT but not in GILZ KO mice (from 22 ± 1 mm to 15 ± 0.5 and from 24 ± 1.5 to 19 ± 0.5 respectively), MPO activity (from 204 ± 3.13 pmol/mg to 73.88 ± 2.63 in WT and from 221 ± 4.46 pmol/mg to 107 ± 5.54 in GILZ KO), the inflammatory response, TGF-β production, and α-SMA deposition in comparison to WT and GILZ KO vehicle groups.

**Conclusion:**

In conclusion, the role of H_4_R and GILZ in relation to glucocorticoids could pave the way for innovative therapies to counteract pulmonary fibrosis.

**Supplementary Information:**

The online version contains supplementary material available at 10.1007/s00011-023-01802-3.

## Introduction

Pulmonary fibrosis is a chronic, progressive disease with an unfavorable prognosis that predominantly affects older individuals [1]. Currently, the incidence and the prevalence of this disease are estimated to be in the range of 0.09–1.30 and 0.33–4.51 per 10,000 people [2]. The etiology of this disease is still unclear, and the underlying causes remain unknown. This pathological condition is characterized by the degeneration of lung tissue resulting in epithelial cell damage, vascular exudation, and leukocyte infiltration into the alveolar spaces [3]. Specific features of this disease are the increase and progressive proliferation of fibroblasts as well as their differentiation into myofibroblasts resulting in an overproduction of collagen and other matrix components such as fibronectin. This accumulation disrupts the architecture of the lung tissue leading to progressive lung degeneration resulting in respiratory failure and, in several cases, to death [3].

Clinical practice for the management of pulmonary fibrosis is based on therapies such as nintedanib, a tyrosine kinase inhibitor, or pirfenidone, a fibroblasts proliferation inhibitor, but not all patients respond efficiently [4–6] and lung transplantation is the ultimate option [1].

Histamine is an important mediator of inflammation and immune-allergic responses, produced by decarboxylation of histidine through the enzyme histidine decarboxylase (HD). Its effects are mediated by the activation of four receptors (H_1_R, H_2_R, H_3_R, and H_4_R) that belong to the family of G protein-associated receptors [7]. Histamine regulates cellular immunity by controlling the production of pro-inflammatory cytokines, chemokines, the expression of adhesion molecules, and the migration of inflammatory cells [8]. In particular, histamine H_4_ receptor (H_4_R), mainly expressed on immune cells and involved in immunomodulatory responses, mediates chronic inflammation of the airways by regulating the activation of CD4^+^ T lymphocytes in the production of Th2-type cytokines [9]. Previous studies demonstrated that this receptor is involved in the pathogenesis of pulmonary fibrosis, and treatment with a highly selective H_4_R antagonist reduces pro-inflammatory and pro-fibrotic markers preventing fibrosis progression in a bleomycin-induced lung injury model [10, 11].

Glucocorticoids (GCs), drugs widely used for the treatment of inflammatory and autoimmune diseases, perform their action by binding to glucocorticoid receptors (GRs) in the cytoplasm [12]. After the interaction, the complex changes its shape. Successively, GRs dissociate from GC/GR complex and translocate into the nucleus, interacting with glucocorticoid recognition elements (GREs) [13] explicating its anti-inflammatory action. GCs are highly effective in counteracting inflammation in several diseases, such as rheumatoid arthritis, asthma, atopic dermatitis, and allergic rhinitis, but despite their large use in clinical practice, GCs show side effects due to chronic exposure [14, 15].

Among the molecules involved in the inflammatory process and regulated by GCs, a key role is played by the glucocorticoid-induced leucine zipper (GILZ), a dexamethasone inducible gene [16]. GILZ regulates proliferation, apoptosis, and differentiation through the modulation of several transcription factors [17]. Specifically, it binds the transcription factor NF-kB, and by inhibiting its activation and nuclear translocation, prevents pro-inflammatory gene transcription.

Annexin A1 (Anx-A1), also known as lipocortin-1, is a protein involved in a variety of pathophysiological processes, including inflammation. Anx-A1 has an anti-inflammatory activity, regulating the trafficking and activation of leukocytes, and it is highly expressed in lung cells [18]. The absence of Anx-A1 in *Anx-A1* null mice increased the degree of inflammation and fibrosis in a bleomycin lung fibrosis model [18]. A correlation between expression of this protein and GCs has been demonstrated, in fact, GILZ deficiency is associated with an early increase of Anx-A1 in LPS-injected mice; moreover, the lack of endogenous GILZ during the resolution of inflammation is compensated by Anx-A1 overexpression, demonstrating that this protein is required for GC induction of GILZ in vivo [19].

Furthermore, GILZ regulates T cell activity modulating the shift from Th1 to Th2 immune phenotypes through the expression of Th2 cytokines, such as IL-4 and IL-10 [20, 21].

Based on the concept that both H_4_R and GILZ drive the differentiation of Th1 into Th2 lymphocytes and that histaminergic drugs and GCs are involved in the resolution of chronic inflammation, we hypothesized a possible interaction between histamine, inflammatory mediators, and GILZ in preventing the progression of lung injury in a murine model of pulmonary fibrosis induced by bleomycin injection.

## Materials and methods

### Drugs and reagents

Compound JNJ7777120 (JNJ; 1-[(5-chloro-1H-indol-2-yl) carbonyl]-4-methylpiperazine), an H_4_R antagonist (Janssen Research & Development, USA), has been used at a concentration of 2 mg/kg of body weight (b.wt.) dissolved in PBS with 1% DMSO. To induce pulmonary fibrosis, bleomycin (Merck-Millipore, Burlington, MA, USA) was used at a concentration of 0.05 IU for each mouse and dissolved in 50 μl of saline. Drug doses and frequency of administration were selected based on previous publications [10].

### Animals

The experimental protocol was carried out on male wildtype (WT) C57BL/6 and their littermate GILZ knock-out (KO) mice [22] weighing ~ 25–30 g and of 8–9 weeks of age. All animals received a standard diet and water ad libitum and were housed at 22 °C with a 12-h light/12-h dark cycle for at least 48 h before the experiments. The study protocol was in accordance with the European Economic Community (86/609/CEE) and the Declaration of Helsinki guidelines on animal experimentation and received approval by University of Florence (Florence, Italy) Animal Ethical and Care Committee and by the Italian Health Ministry (Authorization n° 874/2017-PR). Experiments were approved and performed following the ARRIVE guidelines at the Centre for Laboratory Animal Housing and Experimentation (CeSAL), University of Florence [23].

### Surgery and treatments

A total of 15 C57BL/6 WT mice and 15 GILZ KO mice were used for the study. Ten mice of both groups were injected with a single bleomycin dose (0.05 IU diluted in 50 µl of saline), while the other 5 WT and 5 GILZ KO mice were treated similarly by intra-tracheal injection with 50 µL of saline (negative controls, Naïve) [24].

Prior to the surgical procedure, the animals were anesthetized with zolazepam/tiletamine, 50/50 mg/ml (Zoletil, Virbac Srl, Milan, Italy, at a dose of 50 µg/g b.wt. dissolved in 100 µl of saline i.p.). An incision was made along the line of the neck and the trachea was exposed. The injection of bleomycin solution or saline was done through the gap between two tracheal cartilage rings with a syringe with a needle of 30  gauge.

Starting from day 0, each mouse was treated with continuous infusion of the drug by osmotic micropumps (Alzet, Cupertino, CA, USA). Pumps were filled with 100 ml of PBS, pH 7.4, containing JNJ7777120 (JNJ, 2 mg/kg b.wt.) or vehicle (1% DMSO in PBS, fibrotic control, Vehicle). The micropumps, that release 2.64 μl of solution per day, were implanted subcutaneously into a dorsal poach at day 0 and maintained for 21 days. The mouse's body weight was measured daily during 21 days of treatment in order to exclude any toxic effects of the drug treatment.

### Functional assay of fibrosis

After 21 days of treatment, all mice were subjected to the measurement of airway resistance to inflation, a functional parameter indicative of fibrosis-induced lung stiffness (pressure at airway opening, PAO). The measurement was performed using mechanical ventilation method of a constant volume and respiration rate per min [25, 26]. Briefly, a 22-gage cannula (Venflon 2; Viggo Spectramed, Windlesham, UK, 0.8 mm diameter) was introduced into the trachea of each anesthetized mouse. The mouse ventilation was controlled using a small-animal respirator (Ugo Basile, Comerio, Italy) adjusted to provide a tidal volume of 0.8 ml with a respiratory rate of 20 strokes/min. A high-sensitivity P75 type 379 pressure transducer (Harvard Apparatus Inc., Holliston, MA, USA), with settings of gain 1 and chart speed 25 mm/s, and a recording polygraph (Harvard Apparatus Inc. Edenbridge, UK) with settings of gain 1, chart speed 25 mm/s, were used to register the changes in lung resistance to inflation, i.e., the pressure at airway opening (PAO). Changes in lung resistance to inflation, registered on the polygraph for at least 3 min and expressed as mm, were determined for 40 or more consecutive tracings of respiratory strokes before averaging [24].

### Tissue sampling

After functional measurements of parameters for fibrosis-induced lung stiffness, the animals were killed using anesthetic drugs at a lethal dose. The gross appearance of the lungs was examined, they were excised, and lung wet weight was determined. No macroscopic alterations of this organ were observed. The whole left lungs were excised and fixed by immersion in 4% formaldehyde in PBS for histological analysis. The right lungs were divided into two pieces, one was quickly frozen and stored at – 80 °C, and the other one was submerged in RNA later solution (Thermo Fisher Scientific, MA, USA) for the consecutive extraction of total RNA.

For biochemical analysis, the lung samples were thawed at 4 °C, homogenized on ice in RIPA buffer, and then centrifuged for 30 min at 10,000*g* at 4 °C, unless otherwise indicated. The homogenized supernatants were stored for the following biochemical determinations.

### Myeloperoxidase (MPO) activity determination

Frozen lung samples were weighed and homogenized (10 ml/mg of tissue) in 0.2 M PBS (pH 6), supplemented with protease inhibitors (1 mM PMSF and 1 × Protease Inhibitor Cocktail, Sigma-Aldrich, MO, USA) and were centrifuged at 10,000*g* at 4 °C for 30 min. MPO was measured in the supernatants with a specific immunoassay kit (CardioMPO; PrognostiX, Cleveland, OH, USA), according to the manufacturer’s instructions. Total protein concentration in the lung tissue samples was determined over an albumin standard curve. The results are expressed as pmol/mg of protein. Values are means ± SEM of individual mice from different experimental groups.

### Histological and morphometrical analysis

Paraffin-embedded lung samples were cut into 5 μm thick histological sections. All sections were stained in a single session to minimize artifacts during the staining process. A light microscope was used to register photomicrographs of the histological slides. The microscope was equipped with objectives at different magnifications and connected to a digital camera. Optical density (OD) and surface area were measured using the free-share ImageJ 1.53 image analysis program (https://imagej.nih.gov/ij/) to quantitatively assess the stained sections. Values are expressed as mean ± SEM of individual mice (20 images each) from the different experimental groups.

Lung tissue sections were stained with hematoxylin/eosin in order to quantify the morphometric parameters of smooth muscle layer thickness, a marker of airway remodeling. Microphotographs of small-sized bronchi were randomly recorded digitally. The bronchial smooth muscle layer thickness was measured in the digitized images using the ImageJ analysis software.

Periodic acid-Schiff (PAS) staining was used in order to quantify the bronchial goblet cell number, another marker of airway remodeling. Total bronchial epithelial cells and PAS-stained goblet cells in the bronchial sections were counted, and the goblet cell percentage was calculated [24].

The assessment of lung collagen deposition was obtained by staining the histological sections with a simplified Azan method for collagen fibers [27], omitting azocarminium and orange G to reduce parenchymal tissue background. OD measurements of the aniline blue-stained collagen fibers were performed after selection of a correct threshold to eliminate aerial air spaces and bronchial/alveolar epithelium [28].

### Pro-fibrotic cytokine TGF-β evaluation

The levels of TGF-β, the major profibrotic cytokine involved in fibroblast activation, were quantified and expressed as pg/ml in plasma aliquots (100 μl) using a TGF-β1 mouse ELISA kit (ThermoFisher Scientific, Monza, Italy), following the manufacturer's protocol. Values are expressed as mean pg/μg ± SEM of total proteins determined over an albumin standard curve.

### Immunofluorescence analysis

Immunofluorescence staining was performed as previously reported [3]. Briefly, lung sections were deparaffinized and boiled for 10 min in sodium citrate buffer (10 mM, pH 6.0, purchased from Bio-Optica, Milan, Italy) for antigen retrieval. Successively, the sections were immune-stained with rabbit monoclonal anti-α-SMA antibody (1:200; Abcam, Cambridge, UK) or rabbit monoclonal anti-NF-kB p65 antibody (1:400, Cell Signaling Technology, Danvers, MA, USA) and goat anti-rabbit Alexa Fluor 568-conjugated IgG (1:300; Invitrogen, San Diego, CA, USA). The negative control was the section in which non-immune rabbit serum was substituted for the primary antibodies. DAPI was used as counterstaining, and representative images were acquired by an Olympus BX63 microscope (Milan, Italy) equipped with Olympus CellSens Dimension Imaging Software version 1.6. The markers’ expression was quantified by densitometric analysis of the intensity of the fluorescence signal in digitized images with ImageJ software. Values are expressed as mean ± SEM of the OD measurements (arbitrary units) of individual mice (20 images each) from the various experimental groups.

### Protein extraction and Western blot determination

Lung tissues were lysed with Cell Lysis Buffer 1X (Cell Signaling Technology, MA, USA) containing Protease Inhibitors Cocktail, Phosphatase Inhibitor Cocktail, and 1 mM PMSF (Sigma-Aldrich, MO, USA). The total proteins (100 mg) were evaluated with the use of Micro BCA Protein Assay Kit (Thermo Fisher Scientific, MA, USA), were subjected to 10% SDS-PAGE, transferred to nitrocellulose membranes, and incubated overnight (4 °C) with monoclonal antibody anti-pSMAD3 and SMAD3 (1:1000, Cell Signaling Technology, Danvers, MA, USA), or anti-H_4_R polyclonal antibody (1.5 mg/ml, Abcam, Cambridge, UK) or anti-p65-NF-κB monoclonal antibody (1:1000, Cell Signaling Technology, Danvers, MA, USA) or anti-Anx-A1 polyclonal antibody (1:2000, Invitrogen, IL, USA) in 5% bovine serum albumin (BSA) in TBS-T. After several rinses with TBS-T, membranes were incubated with a secondary antibody (1:2000 in 2% BSA in TBS-T) at RT for 2 h. The loading transfer of equal amounts of proteins was ascertained by reblotting the membrane with an anti-beta actin antibody (Sigma-Aldrich, MO, USA). The bands were visualized by enhanced chemiluminescence (ECL, BioRad, USA) and quantified by densitometric analysis with the ImageJ software.

### Nuclear/cytoplasmic proteins fractionation

Cytosolic and nuclear extracts were prepared as follows: Fresh lung tissue (100 mg) was homogenized at 10% (wt/vol) with a Tissue Lyser (Qiagen, Hilden, Germany) using a homogenization buffer containing 20 mM HEPES (pH 7.9), 1 mM MgCl_2_, 0.5 mM EDTA, 1% Nonidet P-40, 1 mM EGTA, 1 mM DTT, 0.5 mM PMSF, and 1 μl/ml of PIC. Homogenates were centrifuged at 1300 g for 5 min at 4 °C. Supernatants were removed and centrifuged at 16,000 g at 4 °C for 40 min to obtain supernatant containing the cytosolic fraction. The pelleted nuclei were re-suspended in extraction buffer (1/3 volume of the homogenation buffer) containing 20 mM HEPES (pH 7.9), 1.5 mM MgCl_2_, 300 mM NaCl, 0.2 mM EDTA, 20% glycerol, 1 mM EGTA, 1 mM DTT, 0.5 mM PMSF, and 1 μl/ml of PIC and incubated on ice for 30 min, followed by centrifugation at 16,000 g for 20 min at 4 °C. The resulting supernatants containing nuclear proteins were carefully removed. Protein content was determined by the BCA assay, and extracts were stored at − 80 °C until use.

### RNA isolation and qPCR analysis

RNA was isolated from whole tissue fragment using RNA-XPress™ Reagent (MB601, HIMEDIA) and reverse-transcribed using PrimeScript RT reagent Kit, with gDNA Eraser (Perfect Real-Time-TAKARA). Quantitative real-time PCR (qPCR) was performed using the 7300 Real-Time PCR System (Applied Biosystems), SYBR™ Select Master Mix (Applied Biosystems), and TaqMan™ Gene Expression Master Mix (Applied Biosystems). The qPCR TaqMan probes (Applied Biosystems) were as follows: TGF-β Mm00441724_m1, TNF-α Mm00443258_m1, IL-6 Mm00446190_m1, IL-1β Mm00434228_m1, INF-γ Mm00801778_m1, IL-13 Mm00434204_m1*.* Relative expression levels are normalized using βact Mm02619580_g1 and GAPDH Mm99999915_g1.

Primers used in amplification using SYBR® Green method are listed in Table 1.

**Table 1:** Sequences of primers used for SYBR Green qPCR analysis

Gene	Sequence
GILZ	
For	CTCCAGGATTTGGATTTGGA
Rev	CATAGGTCTGGCCCTTACCA
L-GILZ	
For	ACCGCAACATAGACCAGACC
Rev	CACAGCGTACATCAGGTGGT
IL-1β	
For	CTGTGAAATGCCACCTTTTGAC
Rev	CTGCCTGCCTGAAGCTCTTG
IL-4	
For	CGAAGAACACCACAGAGAGTGAGCT
Rev	GACTCATTCATGGTGCAGCTTATCG
IL-6	
For	TCCGGAGAGGAGACTTCACA
Rev	TTTCTGCAAGTGCATCATCG
CCL2	
For	AAGAGGATCACCAGCAGCAG
Rev	TCTGGACCCATTCCTTCTTG
CCR2	
For	TGCCATCATAAAGGAGCCATACCTG
Rev	TGTGGTGAATCCAATGCCCTCT
IL-5	
For	CAATGAGACGATGAGGCTTCC
Rev	CAGTACCCCCACGGACAGTT
GAPDH	
For	GTCGGTGTGAACGGATTTG
Rev	
βact	
For	CGGGCGACGATGCTC
Rev	GGATACCTCTCTTGCTCTGG

### Statistical analysis

The reported data are expressed as mean ± SEM of individual average measures of the different animals per group, for each assay. Significance of inter-group differences was evaluated by one-way ANOVA or two-way ANOVA followed by Bonferroni post hoc test for multiple comparisons. For qPCR analyses, significance is assessed by nonparametric Mann–Whitney *U* test. Calculations were done using Prism 6 statistical software (GraphPad Software, Inc., USA). The results are statistically significant when *p* < 0.05.

## Results

### Expression of GILZ in normal and fibrotic lungs

**Fig. 1:**
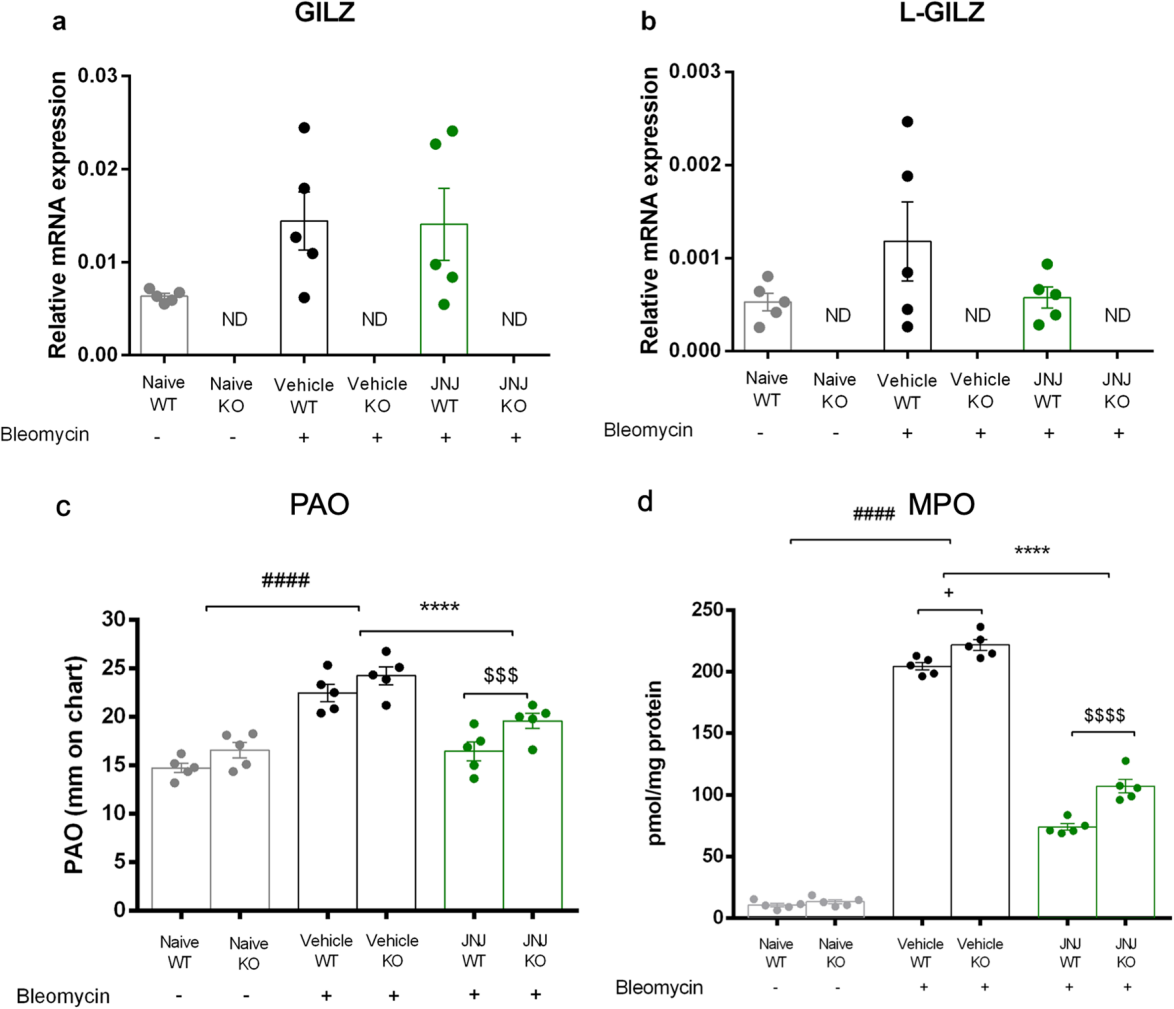
Assessment of mRNA relative expression of **a** GILZ and **b** L-GILZ in normal and fibrotic lungs. Data are mean ± SEM of 5 animals per group. ND, non-detected. **c** Spirometric evaluation of lung resistance to airflow measured by the pressure at airway opening (PAO) analysis (mm on charts). Data are mean ± SEM of 5 animals per group. ^####^*p* < 0.0001 vs Naïve groups; ****p < 0.0001 vs Vehicle groups; ^$$$^*p* < 0.001 vs JNJ WT. **d** MPO levels as markers of leukocyte infiltration. Data are means ± SEM of 5 animals per group. ^####^*p* < 0.0001 vs Naïve; ****p < 0.0001 vs Vehicle; ^$$$$^*p* < 0.0001 vs JNJ WT; + *p* < 0.05 vs Vehicle WT

Among the systems and the molecules involved in the inflammatory process, studies have highlighted the importance of GILZ as a mediator of anti-inflammatory activity [17, 21]. GILZ is expressed not only in the cells of the immune system but also in different tissues, such as epithelium, kidneys, and lungs [21, 29]. Based on this information, we, therefore, performed a preliminary baseline assessment of GILZ levels in the lungs before and after the bleomycin-induced fibrotic process. We evaluated the relative expression levels of GILZ by qPCR analysis in all experimental groups. Figure 1a shows that GILZ slightly increases following the bleomycin-induced fibrotic process as does following the treatment with JNJ, but without showing a statistically significant change. As control, no expression was detected in different groups derived from GILZ KO mice.

Since the GILZ locus can give rise to two different isoforms, GILZ and L-GILZ, both characterized by the presence of the same functional domains and the same function in the inflammatory process [22, 30], we have also assessed the expression levels of L-GILZ isoform. Results show a non-statistically significant increase after the induction of fibrosis with bleomycin as they show no differences in the relative expression levels following JNJ treatment (Fig. 1b).

### Functional assay of fibrosis

Intra-tracheal bleomycin caused a statistically significant increase in airway stiffness, as showed by the clear-cut elevation in the pressure at airway opening (PAO) in the fibrotic positive controls (Vehicle group, 22 ± 1 mm) compared with the non-fibrotic negative controls (Naïve, 14 ± 0.5 mm) for WT mice. Same tendency for GILZ KO mice, PAO of Vehicle group is higher (24 ± 1.5 mm) in comparison to Naïve group (15 ± 0.5 mm).

Compound JNJ caused a statistically significant reduction of airway stiffness (15 ± 0.5 mm) in WT mice. The positive effect of JNJ in reducing PAO is lower in GILZ KO animals (19 ± 0.5 mm) in comparison to WT (15 ± 0.5 mm). Interestingly, PAO is significantly higher in GILZ KO mice treated with JNJ in comparison with WT animals (Fig. 1c).

### Myeloperoxidase activity

Myeloperoxidase (MPO) is a peroxidase enzyme highly expressed in neutrophils and monocytes/macrophage granules in the lung. It is a consistent marker for leukocyte infiltration in inflamed tissues [31]. Very low levels of MPO activity were detected in the naïve groups of both WT and GILZ KO mice (10.48 ± 1.47 pmol/mg and 13.23 ± 1.59 pmol/mg, respectively). Levels of MPO activity increased in the bleomycin-treated mice (Vehicle groups, 204 ± 3.13 pmol/mg in WT and 221 ± 4.46 pmol/mg in KO). A significant decrease of MPO activity was determined after treatment with JNJ in WT and GILZ KO animals in comparison to Vehicle (73.88 ± 2.63 pmol/mg in WT and 107 ± 5.54 pmol/mg in KO). In GILZ KO mice treated with JNJ, MPO activity is significantly higher in comparison with JNJ WT animals (Fig. 1d).

### Effects of JNJ treatment on lung architecture

**Fig. 2:**
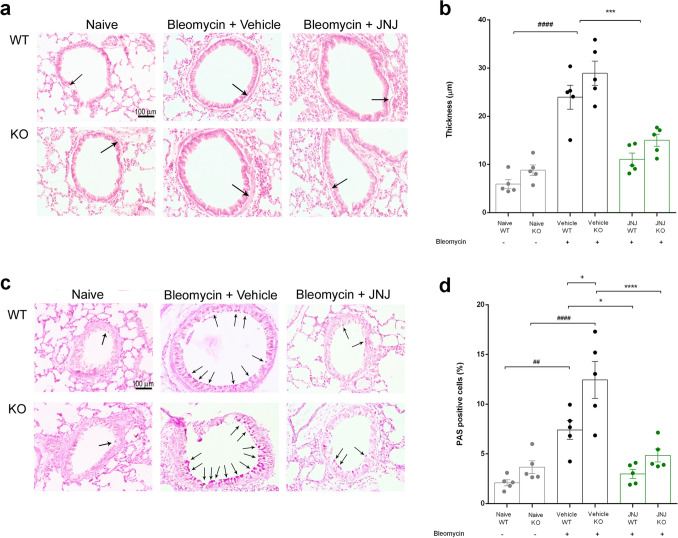
**a** Lung stained with hematoxylin and eosin for the evaluation of smooth muscle layer thickness in the airways highlighted with black arrows (20 × magnification). **b** Dot plot graph shows the thickness in each experimental group. **c** Evaluation of goblet cell presence in lung sections stained with PAS reactive highlighted with black arrows (20 × magnification). **d** The graph represents percentage of goblet cells in the different experimental groups. Data are mean ± SEM of 5 mice per group. ^####^*p* < 0.0001 and ^##^*p* < 0.01 vs Naïve WT and KO, respectively; +*p* < 0.05 vs Vehicle WT; ****p < 0.0001, ***p < 0.001 and *p < 0.05 vs Vehicle WT and KO, respectively

To investigate if an interaction between histamine and GC system is present and what is its role on the bronchial smooth muscle layer, we evaluated the effects of JNJ treatment in WT and GILZ KO lung architecture. Figure 2a, b shows smooth muscle layer thickness in the lungs of different experimental groups stained with hematoxylin–eosin. Animals undergoing stimulation with bleomycin present an increase in the thickness of the peri-bronchial muscle layer; while the treatment with JNJ reduces this damage in a statistically significant way both in WT and GILZ KO animals.

Figure 2c shows lung architecture stained with PAS reactive in order to investigate bronchial mucosa goblet cell hyperplasia. Wild type and GILZ KO mice stimulated with bleomycin present an increased percentage of goblet cells calculated over total bronchial epithelial cells (Fig. 2d). Of note, GILZ KO Vehicle group reports a statistically significant increase compared with the same WT group. Interestingly, JNJ-treated mice report a decrease in the percentage of goblet cells both in WT and in GILZ KO animals, but the latter was statistically more significant than the respective vehicle group.

**Fig. 3:**
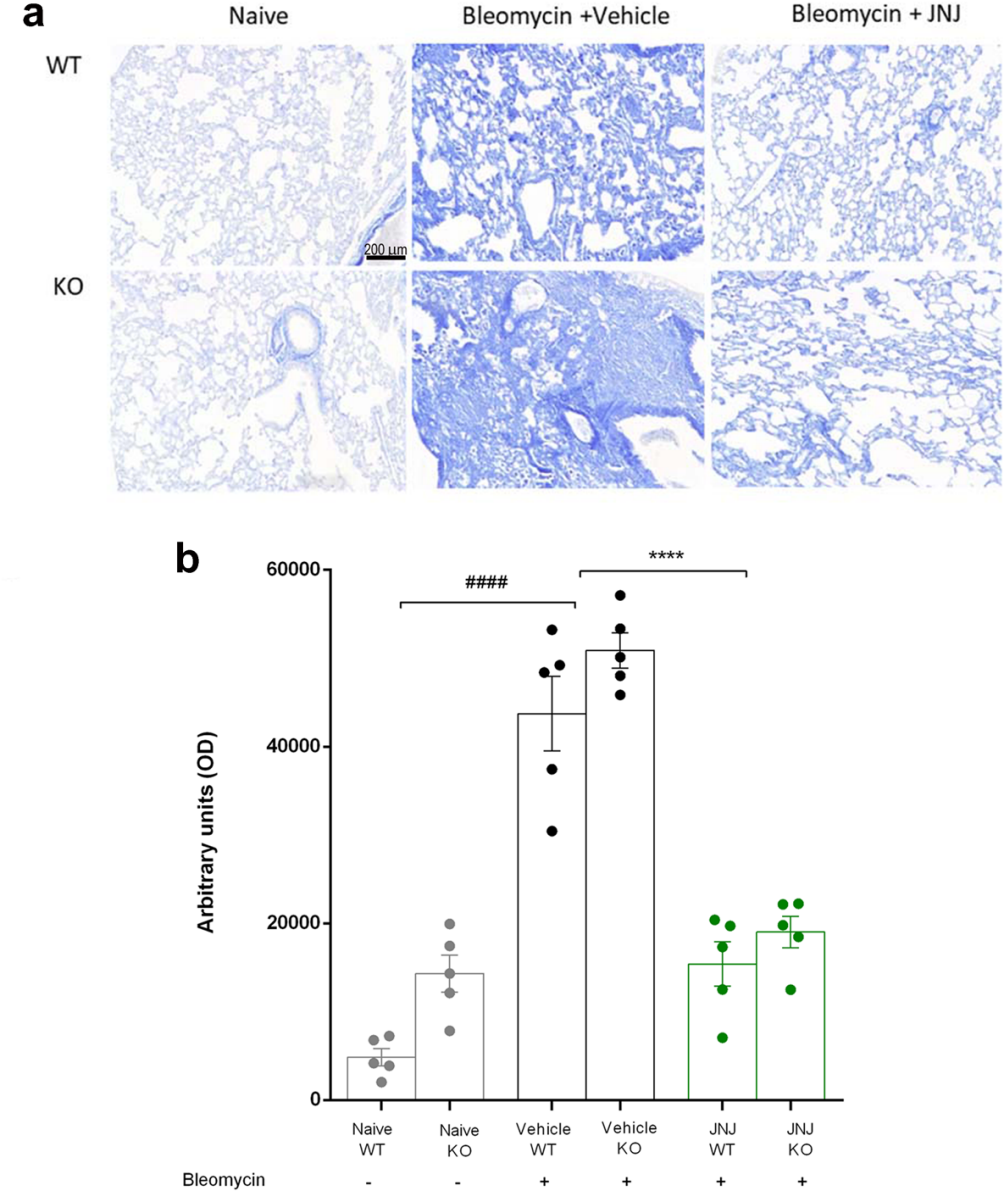
**a** Histopathological evaluation of collagen deposition by Azan-staining lung analysis (magnification 10X). **b** Dot plot graph shows a semi-quantitative measure obtained by computer-aided densitometry analysis in each experimental group. Data are mean ± SEM of 5 mice per group. ^####^*p* < 0.0001 vs Naïve; ****p < 0.0001 vs Vehicle

The results shown in Fig. 3 (a-b) demonstrate that bleomycin administration amplified collagen deposition in interstitial lung spaces with extensive damage of the alveolar structure both in WT and GILZ KO animals. The treatment with JNJ is able to significantly reduce these morphological changes, both in WT and in GILZ KO mice.

### TGF- β signaling pathway

**Fig. 4:**
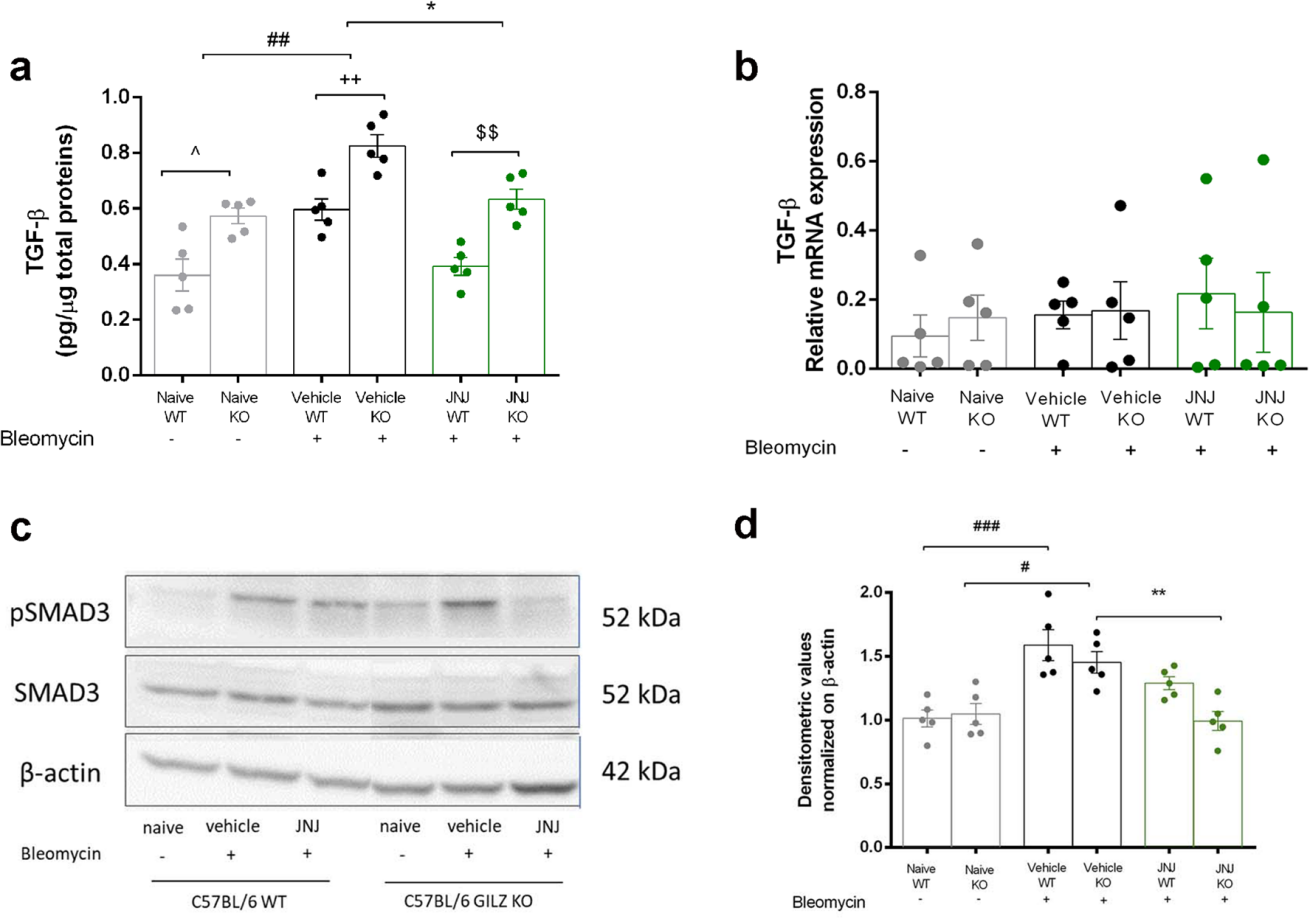
TGF-β signaling pathway evaluation. **a** Assessment of TGF-β production, a pro-fibrotic marker. Values are expressed as pg/µg. **b** Evaluation of mRNA relative expression of TGF-β. **c** pSMAD3 and SMAD3 expression levels were assayed by Western blot. **d** pSMAD3/SMAD3 densitometric analysis normalized on β-actin. Data are mean ± SEM of 5 animals per group. ^###^*p* < 0.001, ^##^*p* < 0.01 and ^#^*p* < 0.05 vs Naïve; **p < 0.01 and *p < 0.05 vs Vehicle; ^++^*p* < 0.01 vs Vehicle; ^$$^*p* < 0.01 vs JNJ WT; ^*p* < 0.05 vs Naïve WT

The increases in TGF-β expression and SMAD pathway signaling lead to the development and the establishment of pulmonary fibrosis [32, 33]. Basal levels of TGF-β are higher in GILZ KO (Naïve) mice in comparison to WT mice. Bleomycin injection increases TGF-β production from 0.36 ± 0.05 to 0.59 ± 0.03 pg/ml in WT mice, and from 0.57 ± 0.02 to 0.83 ± 0.04 pg/ml in GILZ KO mice. Treatment with JNJ for 21 days significantly decreases this production (0.39 ± 0.03 pg/ml in WT and 0.63 ± 0.04 pg/ml in KO). Moreover, TGF-β expression is significantly higher in GILZ KO mice treated with JNJ in comparison with WT animals (Fig. 4a). To confirm our findings, we performed qPCR analysis on lung samples to evaluate whether the GILZ deficiency also affects mRNA expression levels of TGF-β. The data in Fig. 4b show that there are no statistically significant changes between JNJ-treated groups. Still, the trend is consistent with the previous data showed in Fig. 4a. The results indicate that the increase in TGF-β expression observed in GILZ KO mice is not mainly due to a regulation at the transcriptional level.

Phosphorylation of SMAD3 is an effect of TGF-β increase [34], and the expression of SMAD3 and pSMAD3 proteins was evaluated in lung homogenates of WT and GILZ KO mice with Western blot analysis. The ratio between the 2 markers was calculated. WT and GILZ KO bleomycin-treated animals (Vehicle).

presented an increase of pSMAD3/SMAD3 expression in the lung samples, while in JNJ animals, the proteins’ levels decreased both in WT and GILZ KO mice (Fig. 4c, d).

### Lung fibrosis evaluation

**Fig. 5:**
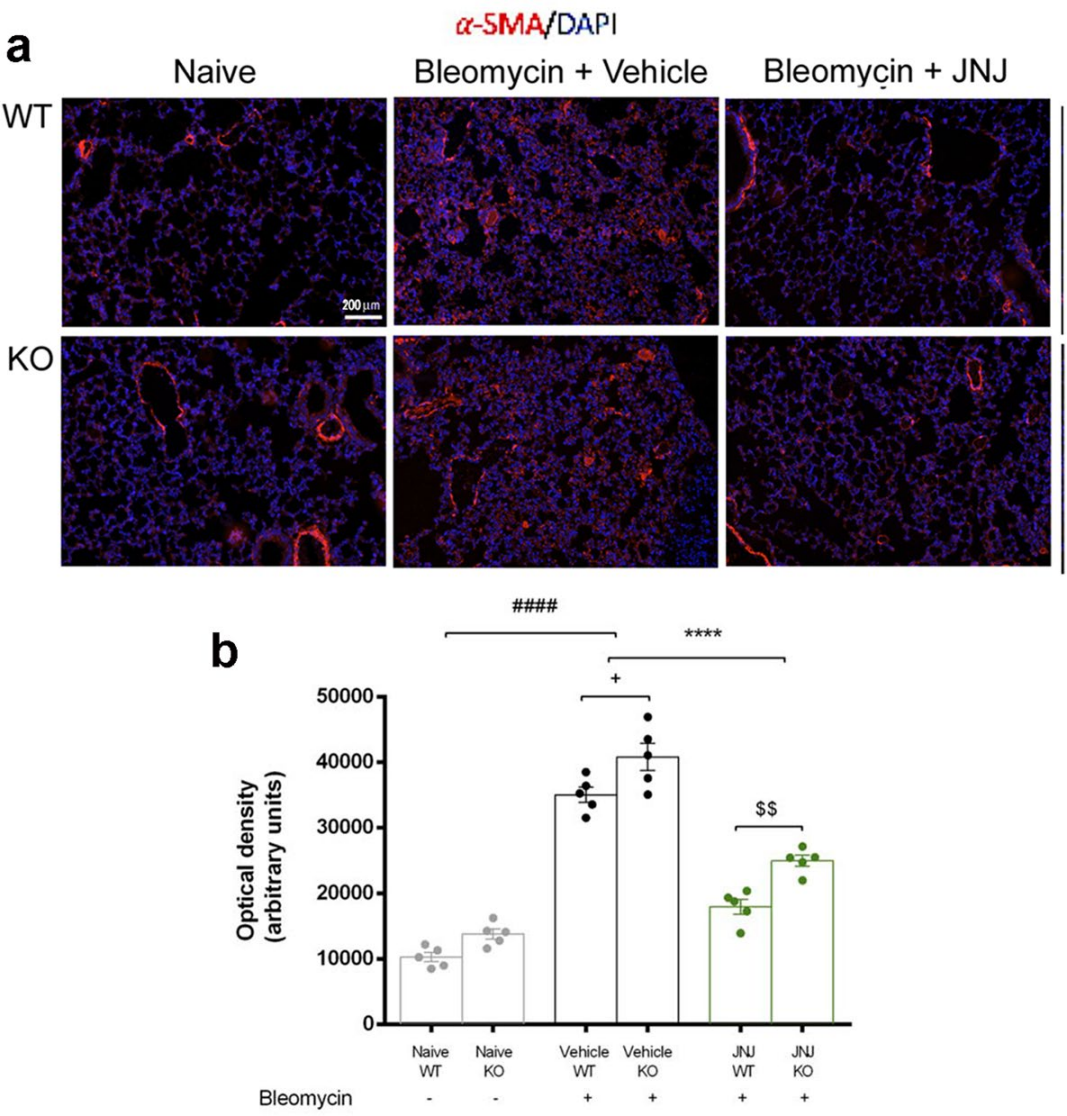
Evaluation of fibroblast differentiation into myofibroblast. **a** Immunofluorescence staining of lung tissue sections labeled with alpha-smooth muscle actin (a-SMA, red) and nuclei (blue) counterstained with DAPI (magnification 10x). **b** Densitometric data are reported as relative optical density (OD) in the different experimental groups. Data are mean ± SEM of 5 animals per group. ^####^*p* < 0.0001 vs Naïve; ****p < 0.0001 vs Vehicle; ^$$^*p* < 0.01 vs JNJ WT and ^+^*p* < 0.05 vs Vehicle WT

TGF-β signaling has been reported to regulate the expression of alpha-smooth muscle actin (α-SMA), a marker of fibroblast activation and myofibroblast differentiation [5]. Immunofluorescence analysis was used to evaluate the expression of α-SMA in the whole tissue, taking care to exclude the perivascular and peribronchial muscular layers. A large increase in α-SMA levels was detected in bleomycin-exposed WT and GILZ KO animals. In the lungs of JNJ-treated WT animals, the levels of α-SMA were drastically reduced. This result clearly indicates that the antagonism of histamine H_4_R reduces fibroblast activation and myofibroblast differentiation in bleomycin-exposed WT mice, while in GILZ KO animals treated with JNJ, the reduction of α-SMA deposition is less marked, indicating that the action of JNJ is partially mediated by GILZ (Fig. 5a, b).

### Expression of NF-kB in lung tissue

**Fig. 6:**
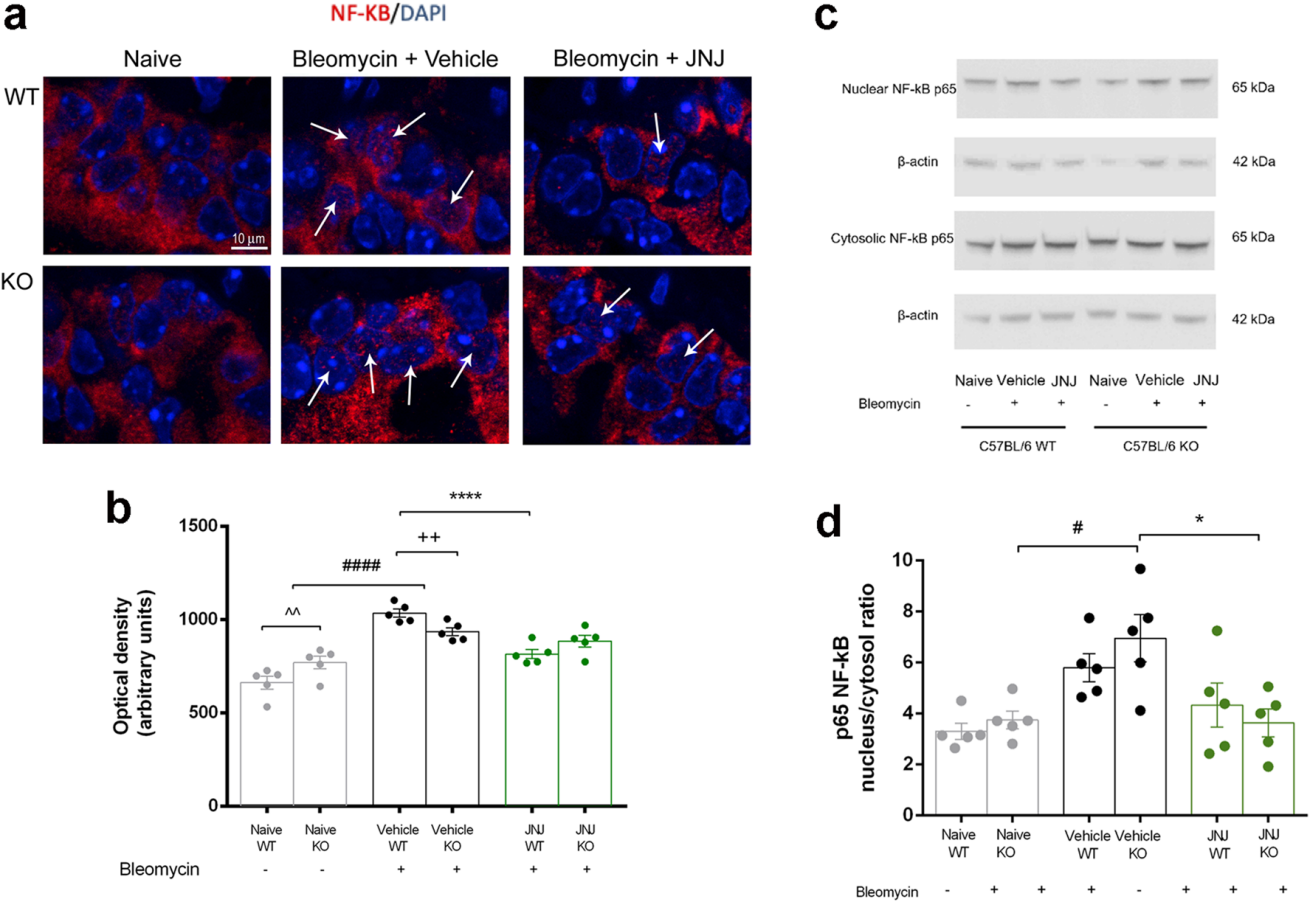
Evaluation of activation of the p65 NF-κB subunit in the lungs. **a** Immunofluorescence staining images of lung tissue sections labeled with p65 NF-κB (red) and nuclei (blue) counterstained with DAPI (magnification 40x). **b** Densitometric analysis of nuclear translocation of p65 NF-κB. Data are reported as relative optical density (OD) in the different experimental groups. **c** Nuclear and cytosolic NF-kB expression levels assayed by Western blotting. **d** Nuclear/cytosolic NF-kB ratio densitometric analysis, normalized on β-actin. Data are mean ± SEM of 5 animals per group. ^#^*p* < 0.05 and ^####^*p* < 0.0001 vs Naïve, *p < 0.05, ****p < 0.0001 vs Vehicle; ^++^*p* < 0.01 vs Vehicle WT; ^^*p* < 0.01 vs Naïve WT

We investigated the effects of JNJ treatment after bleomycin injection on the signaling pathways involved in the activation of NF-κB, since this transcriptional factor plays a pivotal role in inflammation. We observed a significant increase in the nuclear translocation of the p65 NF-κB subunit in the lung of WT and GILZ KO mice stimulated with bleomycin by immunofluorescence analysis. JNJ administration significantly reduced the translocation of the p65 NF-κB subunit from the cytosol to the nucleus in WT mice, while in GILZ KO mice, the treatment with JNJ is less effective because of the severe inflammation of GILZ KO mice (Fig. 6a, b). The results obtained by immunofluorescence are confirmed with nuclear/cytosolic NF-κB ratio by Western blot analysis. The increase of NF-κB nuclear translocation in GILZ KO Vehicle group and the reduction followed by JNJ treatment are detected (Fig. 6c-d).

### *Expression of histamine H*_*4*_*R in lung tissue*

The expression of histamine H_4_ receptor in the lungs was detected by Western blot analysis. The administration of bleomycin causes a statistically significant increase in the H_4_R protein expression in lung homogenates of WT mice, as we previously demonstrated [11].

**Fig. 7:**
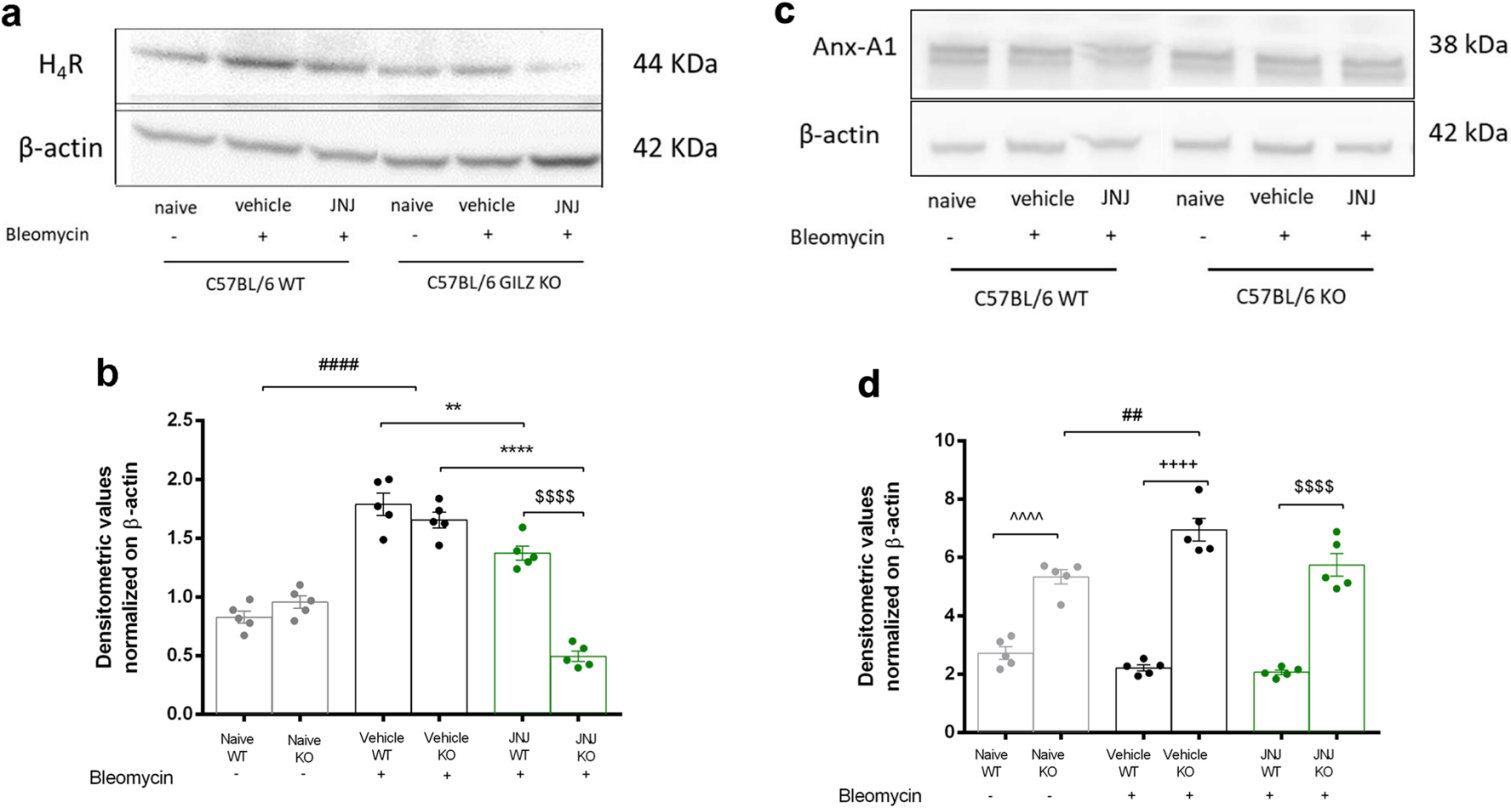
Expression levels of histamine H4 receptor in the lungs, detected by **a** Western blot. **b** Densitometric analysis was normalized on β actin. Data are mean ± SEM of 5 animals per group. ####*p* < 0.0001 vs Naïve; ****p < 0.0001 and **p < 0.01 vs Vehicle; ^$$$$^*p* < 0.0001 vs JNJ WT. **c** Anx-A1 expression levels assayed by Western blot. **d** Anx-A1 densitometric analysis, normalized on β-actin. Data are mean ± SEM of 5 animals per group. ^^^^*p* < 0.0001 vs Naïve WT; ^++ + +^*p* < 0.0001 vs Vehicle WT; ^$$$$^*p* < 0.0001 vs JNJ WT and ^##^*p* < 0.01 vs Naïve GILZ KO

In WT mice, the treatment with JNJ downregulates the expression of H_4_R. In GILZ KO, this downregulation is stronger, probably because the absence of GILZ may exert a modulation of H_4_R expression and the lower expression of the receptor could be translated into a lower efficacy of JNJ (Fig. 7a, b).

### Expression of Annexin A1 in lung tissue

The expression of Anx-A1 in the lungs was detected by Western blot analysis. As already demonstrated [19] and confirmed here, the absence of GILZ causes an increased expression of Anx-A1. In WT mice, its expression is reduced in lungs of animals treated with bleomycin and vehicle (Fig. 7c, d). The administration of JNJ is not able to trigger an increase in Anx-A1 expression, contrary to what was expected [35].

The increased expression of Anx-A1 in GILZ KO mice could exert a compensatory effect that counteracts airway inflammation.

### GILZ absence increases the expression of IL-6 in lung tissue

**Fig. 8:**
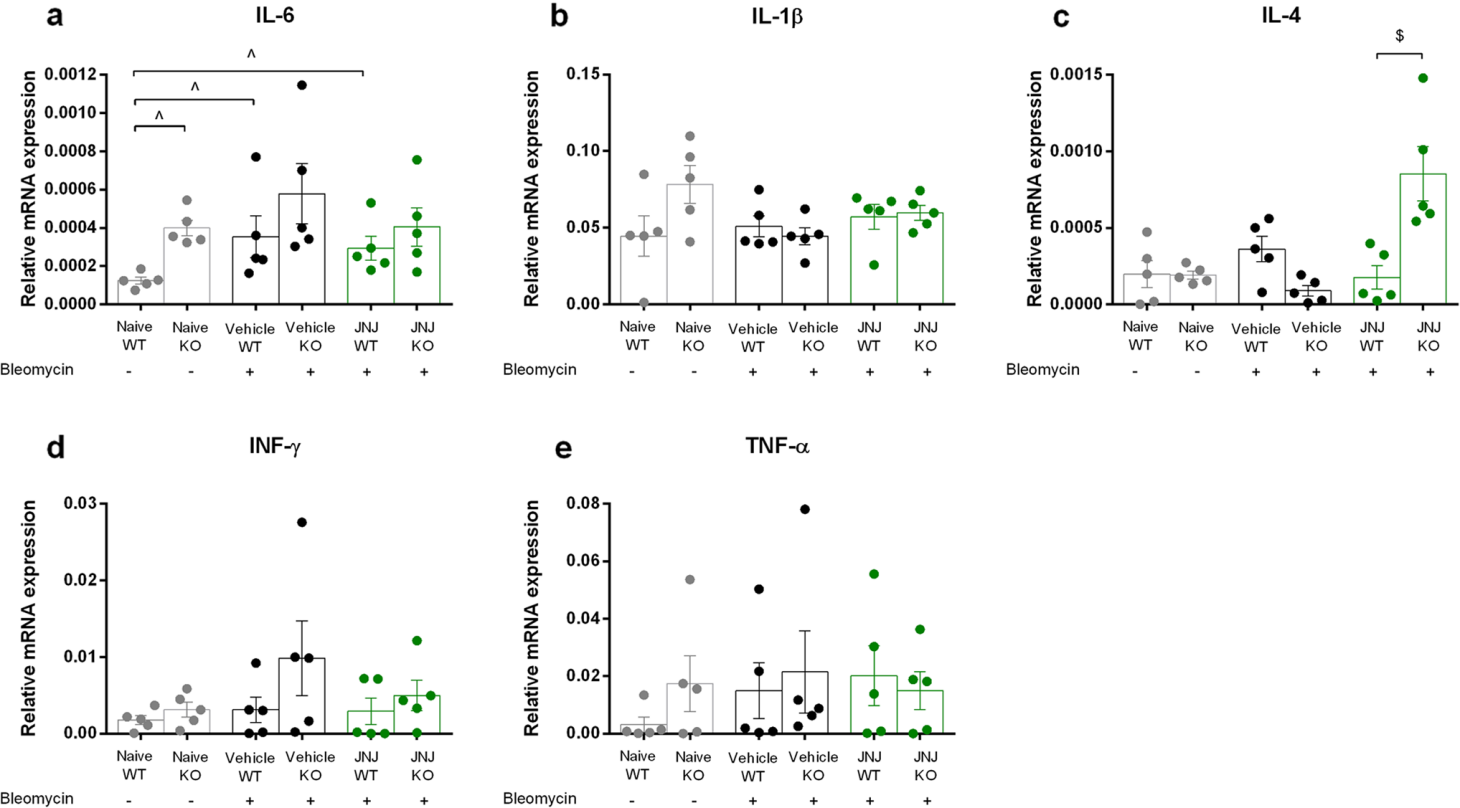
Evaluation of mRNA relative expression of **a** IL-6, **b** IL-1β, **c** IL-4; **d** INF-γ, and **e** TNF-α. Graphs represent mean ± SEM of 5 animals per group. ^*p* < 0.05 vs Naïve, $*p* < 0.05 vs JNJ WT

GILZ is a well-known anti-inflammatory molecule able to inhibit the expression of pro-inflammatory genes and regulate the activation and apoptosis of lymphoid cells [17, 21]. The fundamental points of our analysis were carried out to evaluate the level of damage in fibrotic mice and subsequently to detect whether the treatment with JNJ stimulates the resolution of the inflammatory process. qPCR analysis of mRNA expression levels of pro-inflammatory cytokines shows that there is a significant change in IL-6 mRNA levels of Naïve WT vs. Naïve KO mice (Fig. 8a, column 1 vs. 2) indicating that IL-6 levels are increased in lungs, in the absence of GILZ expression. Comparing the healthy WT with fibrotic WT mice, the level of IL-6 is significantly increased in diseased mice (Fig. 8a, column 1 vs. 3). Lack of GILZ did not significantly change levels of IL-6 in fibrotic lungs (Fig. 8a, column 3 vs. 4). As regards the WT mice treated with JNJ, there is also an important increase compared to the controls (Fig. 8a, column 1 vs. 5). Again, GILZ deficiency did not affect JNJ effect on IL-6 expression levels (Fig. 8a, column 5 vs. 6).

Besides IL-6, we have tested the expression levels of other important cytokines in the inflammatory process, such as IL-1β, INF-γ, and TNF-α, but no other significant difference has been observed among control or treated mice, although the results report variable levels of expression comparing GILZ WT and KO mice, both in the presence of the disease and following treatment (Fig. 8b, d, e).

The pulmonary fibrotic process is strictly connected to the activation and infiltration of T lymphocytes, especially the Th2 subtype. IL-4, IL-5, and IL-13 have been recognized as important mediators of Th2 lymphocytes activity as they represent cytokines with a potential profibrotic role [36–38]. Figure 8c shows how IL-4 mRNA expression levels do not differ when comparing GILZ WT and KO mice, in the control group as in the fibrotic one. To note, a statistically significant increase is however shown in GILZ KO mice after the treatment with JNJ (Fig. 8c, column 6 vs. 5), suggesting that GILZ expression could affect JNJ effect on IL-4 expression. IL-5 and IL-13 expression levels are not detectable through qPCR analysis (not shown).

### The expression levels of CCL2 and CCR2 do not change after induction of fibrosis

Since emerging pieces of evidence indicate that the CCL2/CCR2 axis is involved in fibrotic diseases [39], and since we have previously demonstrated that GILZ deficiency is associated with elevated CCL2 expression and recruitment of CCL2-sensitive immune cells in the liver of GILZ KO mice compared to controls, in a murine model of liver fibrosis [40], we checked the mRNA expression levels of CCL2 chemokine and its receptor, involved in fibrotic progression.

Results show that GILZ deficiency in mice was associated with a slightly elevated CCL2 production in GILZ KO mice after bleomycin-induced fibrosis and after JNJ treatment but no significant differences in mRNA expression levels were found (Supplementary Fig. 1a). The mRNA levels of the CCL2 receptor (CCR2) were subjected to high levels of variability, but not statistically significant alterations between groups were observed (Supplementary Fig. 1b).

## Discussion

The possible interaction between the histaminergic system and glucocorticoid-induced leucine zipper (GILZ) in the genesis and modulation of the inflammatory process was evaluated in a model of bleomycin-induced pulmonary fibrosis after confirming GILZ and L-GILZ presence in lungs (Fig. 1a, b). GILZ and the L-GILZ isoform are expressed in the different GILZ WT groups, while their absence is confirmed in the GILZ KO groups. GILZ slightly increases following disease induction and treatment with JNJ, favoring the hypothesis that GILZ actively participates in the regulation of disease, contributing to an anti-inflammatory effect. L-GILZ instead presents different expression patterns compared to the GILZ isoform, resulting mainly increased in the presence of the fibrotic process, but resulting comparable to the baseline level following drug treatment. These data may imply how L-GILZ is more involved when the fibrotic process is induced, but how it does not actively participate in the resolution of inflammation.

In this research, we used the bleomycin model of lung fibrosis, that is one of the most used experimental system to study lung fibrosis and serves as a widely used preclinical model to test potential therapeutic compounds [3, 10, 11, 24–26, 41–43].

We report here that the treatment with JNJ7777120, an H_4_R antagonist/inverse agonist, ameliorates the functional and structural features of bleomycin-induced lung fibrosis in WT mice, while, in the absence of GILZ gene, the treatment with JNJ seems to be less effective in controlling inflammation and fibrosis, with small differences between the two animal strains, suggesting that other factors could be involved in the regulatory mechanism.

Despite this, the process should be still deeply investigated; however, basing on our results, a synergic interaction between histamine H_4_ receptors and GCs could be hypothesized in order to validate new therapeutic targets for pulmonary fibrosis.

The existing therapeutic approaches to treat lung fibrosis consist of drugs that aim to control the TGF-β signaling pathway and extracellular matrix accumulation: nintedanib, a tyrosine kinase inhibitor that targets multiple tyrosine kinases, and pirfenidone, an agent that reduces TGF-β signaling pathway with anti-inflammatory actions [3, 4, 44].

As demonstrated by the analysis of the functional assay of fibrosis with PAO (Fig. 1c), the administration of bleomycin caused an increase in airway stiffness in Vehicle WT and GILZ KO mice in comparison to the Naïve group; the JNJ treatment reduces this airway stiffness in WT animals, as already demonstrated [10, 11, 42]; in GILZ KO mice, this effect is less evident for the absence of GILZ protein, demonstrating that the effect of JNJ is mediated by GILZ. In fact, the GILZ is a pivotal mediator of the anti-inflammatory effects of GCs [17, 45].

The treatment with bleomycin activates the inflammatory cascade, as clearly underlined by the increase in myeloperoxidase activity. MPO is a peroxidase enzyme highly expressed in neutrophils and monocytes/macrophage granules and it is a consistent marker for leukocyte infiltration in inflamed tissues [35]. In fact, levels of MPO activity increase in the bleomycin-treated mice and a significant decrease in this activity was determined after treatment with JNJ in WT and GILZ KO animals (Fig. 1d). The effect of JNJ in GILZ KO animals is in discrepancy with our hypothesis that the effect of JNJ is mediated by GILZ. A possible explanation is that JNJ acts on the MPO activity through a different pathway that does not involve GILZ; in fact, the treatment with JNJ significantly reduces overall leukocyte infiltration in the lung tissue [35].

To better define the interaction between histamine and GC system on the bronchial smooth muscle layer, we evaluated the effects of JNJ treatment in WT and GILZ KO lung architecture. As already demonstrated in WT mice treated with bleomycin [42], the thickness of peri-bronchial muscle layer, the percentage of goblet cells, and the collagen deposition in interstitial lung spaces are increased; these results are confirmed also in GILZ KO animals. The treatment with JNJ reduces these damages in both groups of animals. All these data demonstrate that the activity of JNJ in the remodeling of the lung architecture acts through a pathway independent from the activity of GILZ (Figs. 2 and 3).

TGF-β and the related signaling pathway are known to play a key role in the development of pulmonary fibrosis [43]. In fact, the increases in TGF-β expression and SMAD pathways signaling lead to the development and establishment of pulmonary fibrosis [32, 33]. As already known [42] bleomycin injection increases TGF-β production and the JNJ treatment decreases this parameter. However, results in Fig. 4b suggest that TGF-β levels are not affected by GILZ at its transcriptional level. Higher production of TGF-β in GILZ KO mice is possibly linked to post-transcriptional modifications or interaction with other molecules involved in the TGF-β signaling pathway. Phosphorylation of SMAD3 is an effect of TGF-β increase; treatment with bleomycin increases pSMAD3 expression in the lungs of WT and GILZ KO animals, while the JNJ treatment decreases the protein levels both in WT and GILZ KO mice, suggesting that the control of fibrosis development is mediated by both H_4_R and GILZ, and further analysis of the action of TGF-β should be carried out.

Moreover, TGF-β signaling has been reported to regulate the expression of α-SMA, a marker of fibroblast activation and myofibroblast differentiation [5]. A large increase of α-SMA levels was detected in bleomycin-exposed WT and GILZ KO animals and the treatment with JNJ drastically reduced the levels of α-SMA. This result clearly indicates that the antagonism of histamine H_4_R reduces fibroblast activation and myofibroblast differentiation in bleomycin-treated WT mice, while in GILZ KO animals treated with JNJ, the reduction of α-SMA deposition is less marked, indicating that the action of JNJ is mediated by GILZ.

Among other transcriptional factors, NF-κB plays a pivotal role in inflammation and it is strictly connected to GILZ expression and activity [46–48]. Our results indicate a significant increase in the nuclear translocation of the p65 NF-κB subunit in the lungs of bleomycin-treated mice by immunofluorescence and Western blot analyses. JNJ administration reduced the translocation of the p65 NF-κB subunit from the cytosol to the nucleus in WT mice, with a less evident effect observed in GILZ KO mice, consistent with the fact that GILZ inhibits NF-κB nuclear translocation [46].

As already known, histamine is implicated in the pathophysiology of several inflammatory and immunological mechanisms, acting in both acute and chronic phases of inflammatory processes. The administration of bleomycin causes a statistically significant increase in the H_4_R protein expression in lung homogenates of WT mice, and the treatment with JNJ downregulates the expression of H_4_R, as we previously demonstrated [11]. In GILZ KO, this downregulation is stronger (Fig. 7a, b), probably because JNJ in physiological conditions can interact with GILZ, which can modulate the expression of H_4_R; the lower expression of the receptor could be translated into a lower efficacy of the drug. H_4_R and GILZ are present in CD4^+^ cells and they cooperate in the resolution of inflammation. Anx-A1 is an important protein for the resolution of the inflammatory response [19] and a potent modulator of leukocyte trafficking/transmigration in acute and chronic inflammation [18]. The Anx-A1 protein is highly expressed in the airways, both in human/animal alveolar macrophages and epithelial cells [18]. As already demonstrated [19] and confirmed here, the absence of GILZ causes an increased expression of Anx-A1 (Fig. 7c, d). In WT mice, its expression is reduced in lungs of animals treated with bleomycin and vehicle. The administration of JNJ is not able to trigger an increase in Anx-A1 expression, contrary to what was expected [35]. The increased expression of Anx-A1 in GILZ KO mice could exert a compensatory effect that counteracts airway inflammation.

Knowing that GILZ is an inflammatory mediator, studying whether its absence could influence the immune response and the release of inflammatory mediators was an important point. IL-6 cytokine is produced by immune cells in injured tissues and could affect immune reactions, especially the inflammatory phase contributing to the switch to a reparative environment during the resolution of wound healing [49]. IL-6 was upregulated in GILZ KO mice, either in treated and not treated mice (Fig. 8a). To note, this result confirms the anti-inflammatory effects of GILZ already shown with the reduction of α-SMA deposition and collagen production, aforementioned. As shown in Fig. 8b, d, e, other pro-inflammatory cytokines, such as IL-1β, INF-γ, and TNF-α, do not show important differences when comparing GILZ WT and KO mice. Their slight increase in GILZ KO mice and pathological conditions, however, could still be related to the lack of GILZ in the presence of an inflammatory environment. The same can be said about IL-4 expression levels (Fig. 8c). IL-4 is known as a profibrotic mediator and its increase in JNJ-treated GILZ KO mice may confirm that inflammatory cells accumulate in the damaged tissues and produce cytokines able to activate fibroblasts and consequently the fibrotic process [37]. JNJ significantly increases the mRNA expression of IL-4 in GILZ KO mice, probably because it increases the M2-Type macrophage development, and subsequently the production of TGF-β that increases the pro-fibrotic processes, especially if GILZ expression is absent. Probably, this happens because the absence of GILZ affects the anti-inflammatory action of JNJ, indicating an interaction between the GC pathway and the histaminergic system. However, the slight effect of H_4_R antagonist treatment on cytokines RNA expression might be ascertained to the fact that other regulatory mechanisms are involved in the mechanisms of inflammation and fibrosis.

We also evaluated the presence of variations in the expression of the CCL2 and CCR2, a chemokine and its receptor essential for regulating T cell infiltration [36]. Previously, we studied the role of GILZ in a model of liver fibrosis (LF) showing how GILZ controls LF development by regulating CCL2-dependent leukocyte trafficking [40]. In the analysis carried out on the fibrotic lungs of mice treated with JNJ, no major differences are found in CCL2 and CCR2 expression levels, suggesting that the role of CCL2 on T lymphocyte migration may not be preponderant when compared to other inflammatory signals (Supplementary Fig. 1).

The results reported in this study should be considered in light of some limitations. Among the limitations of the study that certainly occurred is that of the sample size, for some determinations could be a limiting element in the achievement of statistically significant variations which can be considered reproducible.

This research has allowed advancement of knowledge of the mechanisms of inflammation and fibrosis development, with regard to the interactions between the histaminergic system and the production of pro- and anti-inflammatory cytokines. Some actions of GCs on inflammation are in common with the H_4_R receptor ligands. Therefore, the results of this research have led to the validation of new histaminergic drugs useful for enhancing the therapeutic effects of GCs with a significant reduction in adverse effects. The clinical development of this research could lead not only to a scientific impact for the advancement of knowledge but also certainly to social and economic implications, given the widespread cynical use of GCs.

## Conclusions

The results show that the compound JNJ7777120, a histamine H_4_R antagonist, reduces the functional and the structural features of bleomycin-induced lung fibrosis in WT mice and that it is less effective in reducing these effects in GILZ KO mice. It is reasonable to hypothesize that histamine H_4_R antagonists could represent a new valuable therapeutic strategy for the treatment of the inflammatory and fibrotic process. Furthermore, the use of a mouse model with deletion of GILZ allowed us to demonstrate that GILZ acts as an anti-inflammatory therapeutic protein in bleomycin-induced lung fibrosis model. In fact, inflammation and lung fibrotic process increased in the absence of GILZ protein. In conclusion, the characterization of the role of histamine, H_4_R receptor and GILZ could pave the way for innovative therapies for various chronic inflammatory diseases (allergy, asthma, chronic itch, intestinal disorders) and autoimmune diseases (arthritis. rheumatoid, multiple sclerosis).

### Supplementary Information

Below is the link to the electronic supplementary material.

Supplementary file1 (TIF 274 KB)

Supplementary file2 (TIF 1516 KB)

Supplementary file3 (TIF 1609 KB)

Supplementary file4 (TIF 1693 KB)

Supplementary file5 (TIF 838 KB)

## Data Availability

All relevant data are reported in the manuscript. The raw datasets used and analyzed during the current study are available from the corresponding author upon request.
