# Supplementary figures and images for: Role of histamine H4 receptor in the anti-inflammatory pathway of glucocorticoid-induced leucin zipper (GILZ) in a model of lung fibrosis
Source: Inflamm Res. 2023 Oct 10;72(10-11):2037–52. doi: 10.1007/s00011-023-01802-3 (PMC10611623; doi:10.1007/s00011-023-01802-3)

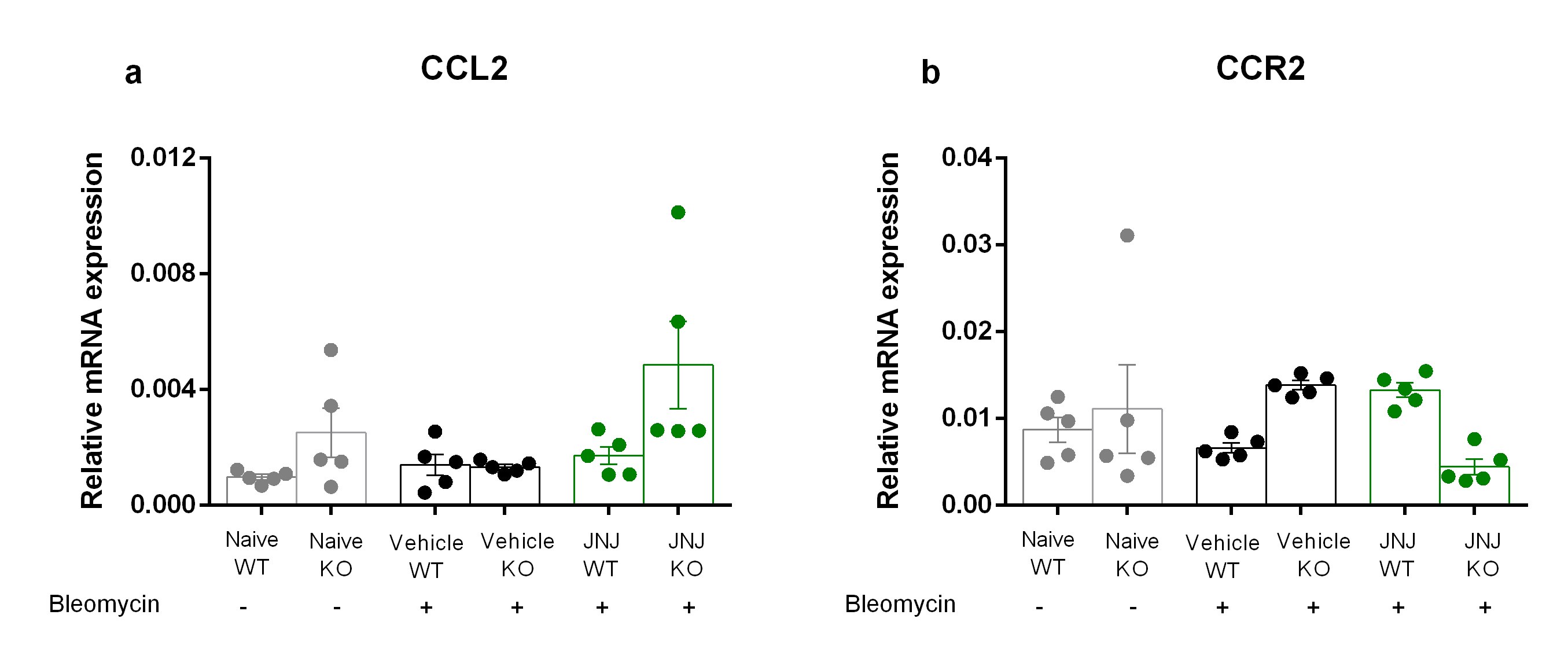

Supplement: Supplementary file 1 — Supplementary file1 (TIF 274 KB) [file 11_2023_1802_MOESM1_ESM.tif]

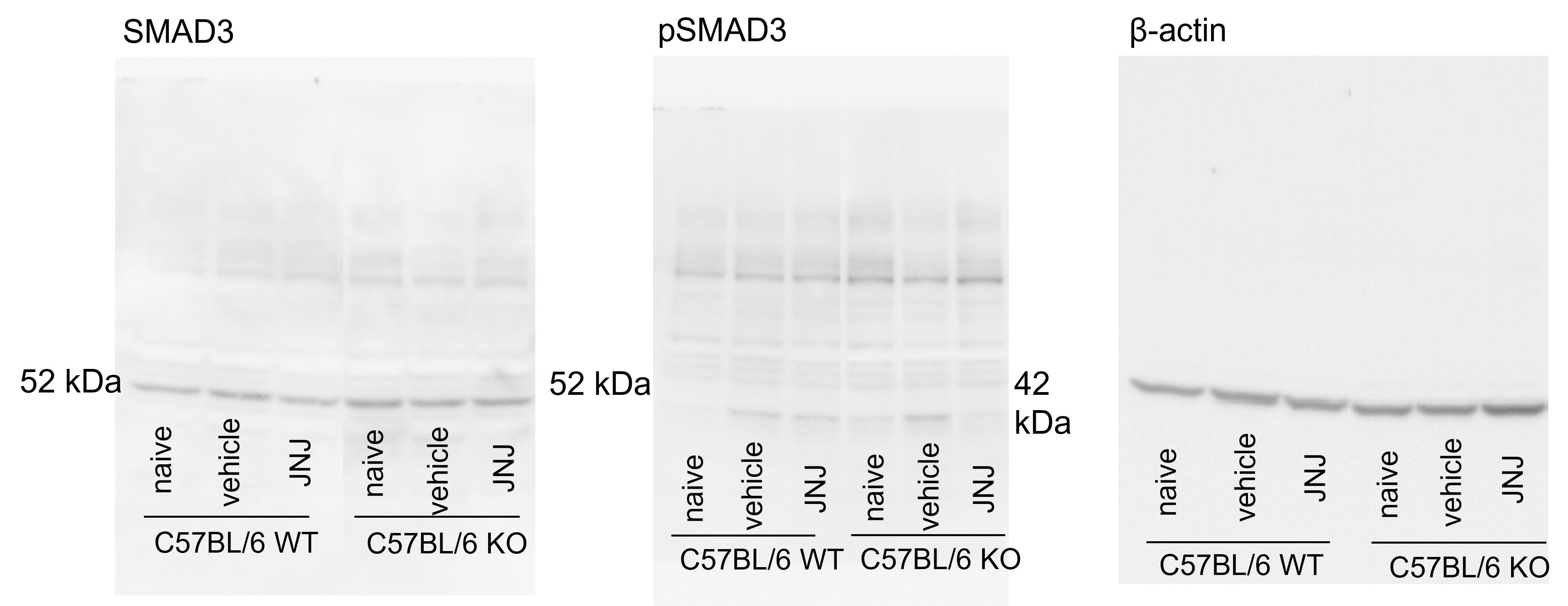

Supplement: Supplementary file 2 — Supplementary file2 (TIF 1516 KB) [file 11_2023_1802_MOESM2_ESM.tif]

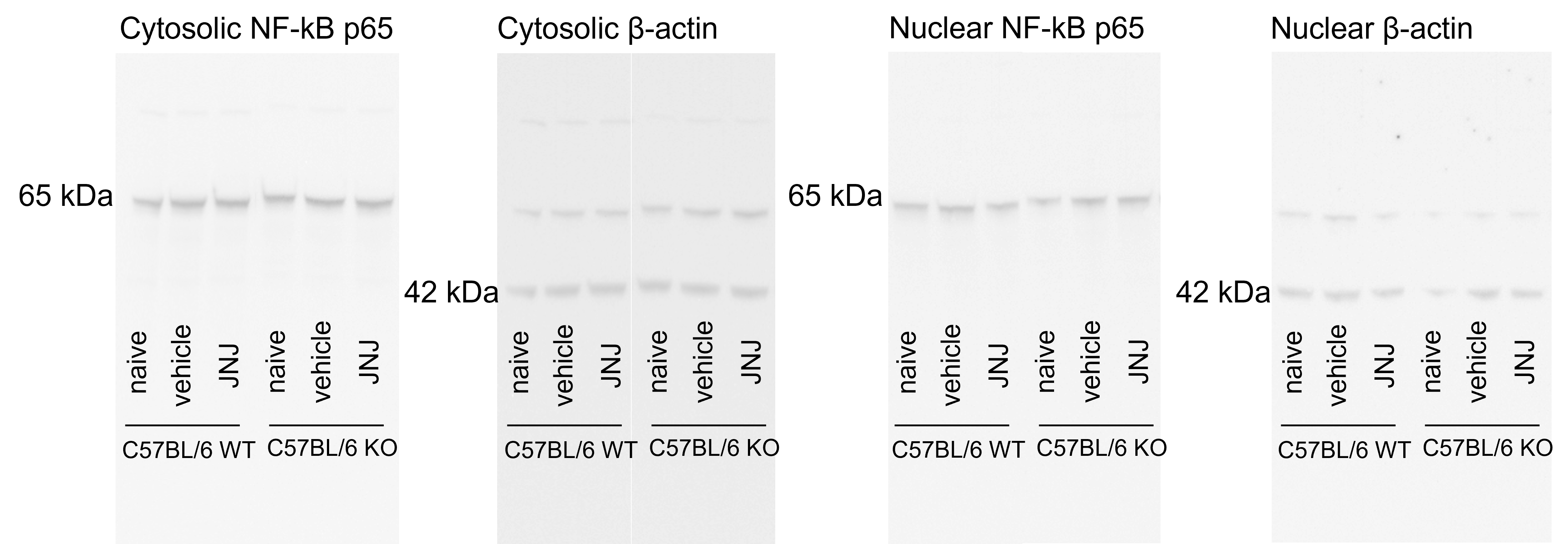

Supplement: Supplementary file 3 — Supplementary file3 (TIF 1609 KB) [file 11_2023_1802_MOESM3_ESM.tif]

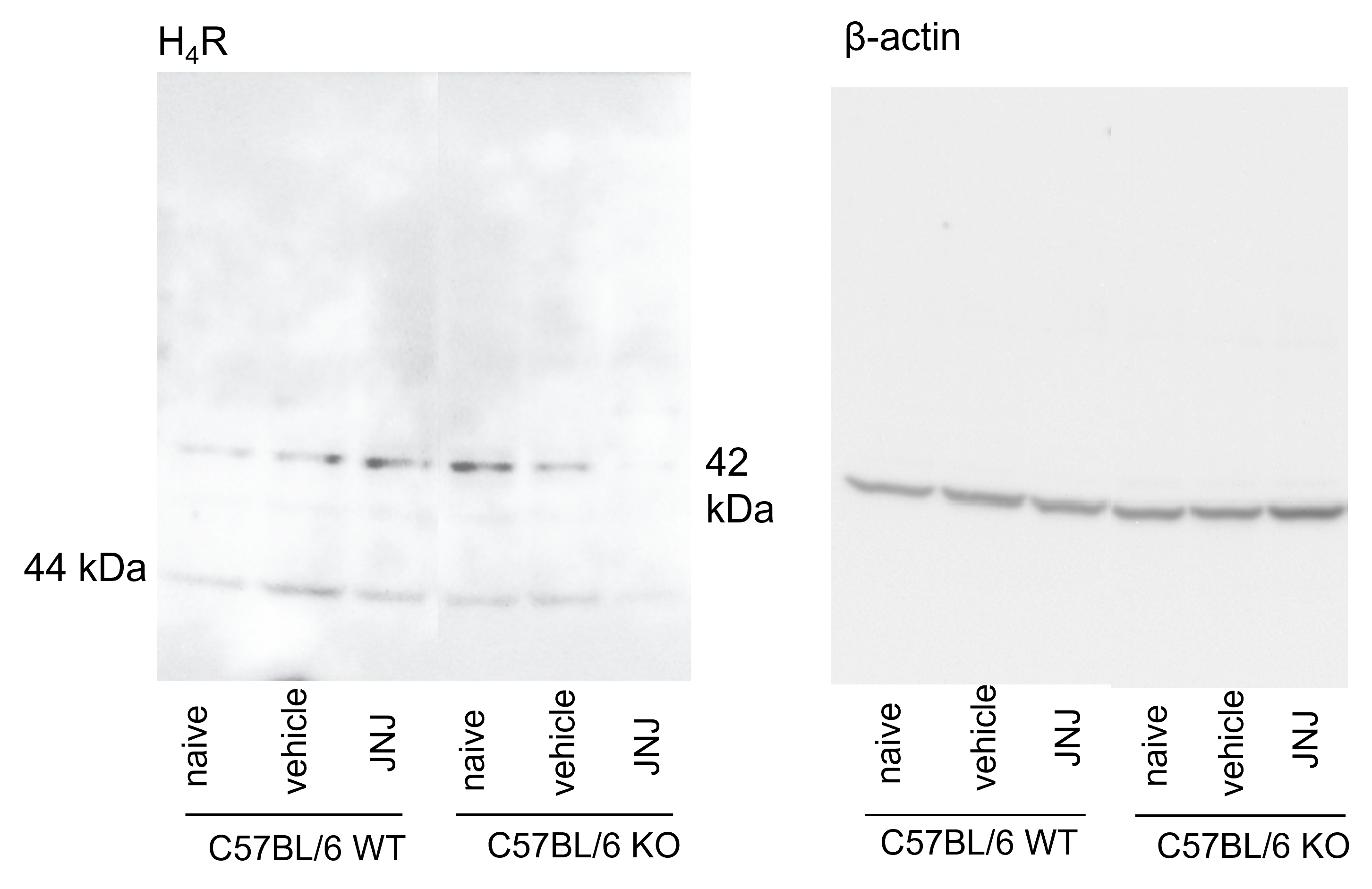

Supplement: Supplementary file 4 — Supplementary file4 (TIF 1693 KB) [file 11_2023_1802_MOESM4_ESM.tif]

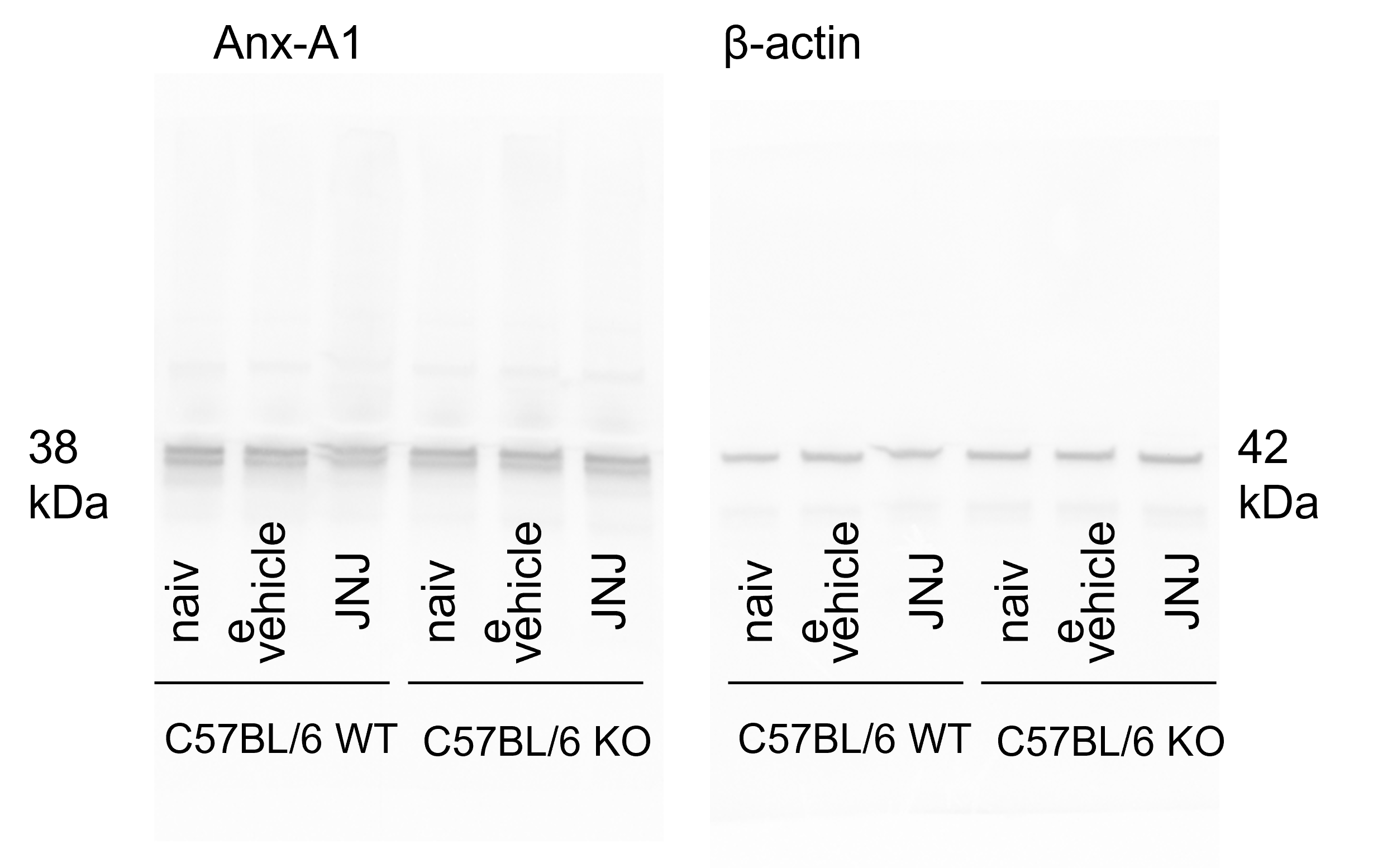

Supplement: Supplementary file 5 — Supplementary file5 (TIF 838 KB) [file 11_2023_1802_MOESM5_ESM.tif]
